# Regenerative Medicine for Neurological Disorders

**DOI:** 10.1100/tsw.2010.39

**Published:** 2010-03-16

**Authors:** Dong-Hyuk Park, David J. Eve, Yong-Gu Chung, Paul R. Sanberg

**Affiliations:** ^1^ Center of Excellence for Aging and Brain Repair, Department of Neurosurgery, University of South Florida College of Medicine, Tampa, FL, USA; ^2^ Department of Neurosurgery, Korea University Medical Center, Korea University College of Medicine, Seoul, South Korea, Republic of Korea

**Keywords:** alternative medicine, ASNTR, neuroscience, regenerative medicine, stem cell, transplantation

## Abstract

The annual meeting of the American Society for Neural Therapy and Repair (ASNTR) has always introduced us to top-notch and up-to-date approaches for regenerative medicine related to neuroscience, ranging from stem cell–based therapy to novel drugs. The 16th ASNTR meeting focused on a variety of different topics, including the unknown pathogenesis or mechanisms of specific neurodegenerative diseases, stem cell biology, and development of novel alternative medicines or devices. Newly developed stem cells, such as amniotic epithelial stem cells and induced pluripotent stem cells, as well as well-known traditional stem cells, such as neural, embryonic, bone marrow mesenchymal, and human umbilical cord blood–derived stem cells, were reported. A number of commercialized stem cells were also covered at this meeting. Fetal neural tissues, such as ventral mesencephalon, striatum, and Schwann cells, were investigated for neurodegenerative diseases or spinal cord injury. A number of studies focused on novel methods for drug monitoring or graft tracking, and combination therapy with stem cells and medicine, such as cytokines or trophic factors. Finally, the National Institutes of Health guidelines for human stem cell research, clinical trials of commercialized stem cells without larger animal testing, and prohibition of medical tourism were big controversial issues that led to heated discussion.

## INTRODUCTION

According to the *Oxford English Dictionary*, “regenerate” means as follows: 1. regrow (new tissue), 2. bring new and more vigorous life to (an area or institution). Recently, many researchers have sought for regenerative therapies that can restore injured cells or replace lost cells with new ones in the human body with permanent injury or intractable disorders. This concept in regenerative medicine is somewhat different from the role of traditional modern medicine, which aims to remove the causes of diseases to cure or stop progression and consequently improve symptoms. There are a number of diseases that cannot be cured with current treatments. Additionally, even if a certain disease could be cured by traditional treatments, lost or damaged cells might not be repaired or regenerated by the treatment and surviving patients would then be expected to live with some sequale. Therefore, organs or tissues lacking the capability of self-regeneration, such as the central nervous system (CNS), are major targets for regenerative medicine.

Currently, neural regeneration has been one of the most actively developing fields in regenerative medicine. The annual meeting of the American Society for Neural Therapy and Repair (ASNTR) has focused on the latest research trends in neural regenerative medicine. The 16th annual meeting of the ASNTR, held in Clearwater, Florida in 2009, presented a varied field, including studies targeting neural repair. Data presented at the meeting may be relatively raw and incomplete, but they are also both cutting-edge and novel. Moreover, a number of topics have been expanded on over a couple of meetings and, consequently, published elsewhere with accumulated data, following their introduction at the ASNTR annual meeting. Therefore, we thought that an annual review of the proceedings at the annual ASNTR meeting would help us to catch up with accumulative scientific evidence in the latest research scenes and knowledge of neural regenerative medicine. We have therefore written this review of the presentations at the 2009 ASNTR meeting and are providing a contextual perspective using similar papers published recently in *TheScientificWorldJOURNAL*. It is worth noting that the proceedings are not rigorously peer reviewed and are unlikely to be published in their presented form without additional studies and data, but we feel that they can still provide an important insight into the latest research targeting neural repair.

At the 16th ASNTR annual meeting, most topics could be divided into three groups: studies directly related to specific neurological disorders, reports focusing on the exploration of various cells for transplantation or biological properties, and issues dealing with cell optimization to achieve a better efficiency. With respect to disease categorization, there was an abundance of studies looking at different methods for approaching neurological diseases, ranging from cell-based therapy through administration of trophic factor gene transfected vectors, to novel drugs or dietary supplementation. However, basic issues such as cell characterization/preparation and neural pathophysiology were still noted because they are critical backgrounds for future pre- and clinical studies. In this review, we introduce and discuss a number of interesting topics presented at the 16th ASNTR meeting according to the above three categories.

## PARKINSON'S DISEASE

Parkinson's disease (PD) is one of the major topics at the ASNTR annual meeting (see Table 1). Although the number of presentations focusing on PD slightly decreased at the 2009 meeting compared with previous meetings, it still was a major subject (about 26%). Pathophysiology about PD itself and treatment-induced side effects, such as drug-induced dyskinesia, are still hot issues in the research field of PD. Monahan et al.[1] reported that prenatal toxin exposure may affect the development of the nigrostriatal pathway. They observed reduced process extension of tyrosine hydroxylase (TH)–immunoreactive neurons in the culture of prenatal lipopolysaccharide-exposed ventral mesencephalon (VM) and lateral ganglionic eminence cells. Nevalainen et al.[2] showed that serotonergic nerve fibers converted L-dopa (Levo-dopa) into dopamine in the absence of dopaminergic nerve fibers. In this study, L-dopa was locally applied into the dopamine or dopamine/serotonin-denervated striatum of rats, and the conversion into dopamine was observed with electrochemical methods. In dopamine-denervated striatum, an increase in extracellular dopamine concentration was seen when stimulating terminals with potassium chloride after local L-dopa administration, whereas no such increase was detected in the dopamine/serotonin-denervated striatum.

In *TheScientificWorldJOURNAL*, Quiroz et al.[3] looked at the role of presynaptic adenosine receptors in cortical neurotransmission to the striatum, and how these receptors may modify the response of neurons to neurotransmitters such as dopamine. This could be relevant to our understanding of PD as well as neuropsychiatric disorders.

**TABLE 1**
**Distribution of Major Topics in 2009 ASNTR Meeting**

| | No. of References |
|---|---|
| Parkinson's disease | 26 |
| Mechanisms and characterization | 10 |
| Stroke/ischemia | 9 |
| Alzheimer's disease | 8 |
| Spinal cord injury | 8 |
| Cell preparation | 8 |
| Aging | 6 |
| Amyotrophic lateral sclerosis | 4 |
| Huntington's disease | 4 |
| Pain | 2 |
| Traumatic brain injury | 2 |
| Others | 12 |

A second study published in *TheScientificWorldJOURNAL*[4] investigated an improved *in vitro* model of PD. The use of a localized injection of 6-hydroxydopamine (6-OHDA) to the cell surface of a VM organotypic slice culture was discussed. This ties in well with the increased pathophysiological interest in the medial forebrain bundle (MFB) as a PD animal model. Terminal 6-OHDA lesions of the rat striatum more closely represent the early and middle stages of human idiopathic PD, whereas the MFB lesion corresponds to a more complete destruction of the dopaminergic nigrostriatal pathway as seen in the later PD stages. Timmer et al.[5] investigated the role of motor training prior to lesion in this animal model. They found that trained animals with MFB lesions showed better behavioral levels 4 months postlesion compared to untrained animals, whereas the training levels had no influence in striatally lesioned animals. More pronounced deficits in the MFB-lesioned groups were also observed compared to the terminal-lesioned group. By contrast, Lieu et al.[6] studied the role of interhemispheric nigrostriatal connections for the genesis of drug-induced dyskinesia in PD. Interhemispheric nigrostriatal connections have been reported to constitute 2–10% of the nigrostriatal pathway in mammals. They used a unilateral injection of 6-OHDA into the striatum to produce a striatal partial lesion and into the MFB to produce a complete lesion. Exposure to L-dopa caused significant dyskinesias in all MFB-lesioned animals, while striatal partial lesions showed no apparent dyskinesias. Of interest, an immunohistochemical analysis revealed that interhemispheric nigrostriatal neurons were preserved in the striatal partial-lesioned animals, which did not develop drug-induced dyskinesia.

Another common model for PD is the use of 1-methyl-4-phenyl-1,2,3,6-tetrahydropyridine (MPTP) treatment. Behrouz et al.[7] showed that parkin up-regulation may contribute to the recovery of hypothalamic tuberoinfundibular dopamine neurons following MPTP treatment, rather than nigrostriatal dopamine neurons in the MPTP mouse model. Boger et al.[8] demonstrated both genotypic and sex differences in response to potassium-evoked dopamine release, and suggest that at least at the younger age studied, male mice were more affected by the glial cell–derived neurotrophic factor (GDNF) gene deletion than female mice, in terms of dopamine neurotransmission using the GDNF knock-out mouse model. The male mice exhibited a delayed dopamine response to potassium compared to female mice, which displayed an increased response in the microdialysis paradigm used.

The potential use of diverse cells and/or tissues for transplantation is still being actively investigated. Although fetal mesencephalic tissue is a major target of cell-based therapy for PD patients, various stem cells, such as embryonic stem cells (ESCs), neural stem cells (NSCs), and human umbilical cord blood

(hUCB) stem cells, have emerged as alternative cell sources that can be applied to differentiate into or protect dopamine neurons, or exert neurotrophic effects. In the studies focusing on the transplantation of fetal mesencephalon, it was shown that neither VM nor striatal grafts, which were derived from GDNF gene-deleted tissue, survived for longer than 3 months in a normal mouse brain[9]. This finding suggests that GDNF is important for long-term survival of grafted nigrostriatal cografts. Sladek et al.[10] revealed that injection of the GDNF gene-transfected viral vector into the striatum with transplantation of fetal VM into the substantia nigra (SN) elicited neuritic outgrowth to the caudate nucleus. Astrocytes were also shown to play a key role in guiding nerve fiber growth from transplanted VM neurons in an *in vitro* experiment using organotypic slice cultures from E14 VM[11]. Steece-Collier et al.[12] demonstrated the role of postsynaptic striatal spiny neurons for dopamine terminal replacement therapy. They found that dopamine grafts implanted into severely parkinsonian rats with a normal compliment of dendritic spines protected by nimodipine showed enhanced behavioral efficacy and decreased L-dopa–induced dyskinesias, as well as increased TH-positive fiber density compared to dopamine neuron–grafted rats with dendritic spine loss.

Another study revealed that the most important thing for restoration of function following transplantation of dopamine neurons in PD was not dopamine levels, but dopamine terminals, using dopamine neurons from dopamine transporter knock-out mice (DATKO)[13]. Although DATKO grafts causing high and diffuse extracellular dopamine levels provided functional recovery even in the presence of abnormally high extracellular dopamine levels, wild-type dopamine neurons showed advantages to restore function and reduce L-dopa–induced dyskinesia.

Among the studies with stem cells, Newman et al.[14] reported on the isolation and culture of CD133 stem cells from hUCB, followed by their transplantation into a 6-OHDA rat model. Intrastriatal transplantation of CD133 stem cells significantly improved behavioral motor functions similar to that seen with a mononuclear fraction of hUCB. With the extension of this study, Kelly et al.[15] also revealed that intrastriatal implantation of hUCB-derived mononuclear fraction and neural-induced CD133 stem cells produced significant improvements in neurological behavioral tests compared to transplantation of human ESCs in a 6-OHDA rodent model. Furthermore, the hUCB-derived stem cell grafts appeared to be more widely distributed and migrated further into the contralateral side than human ESCs. By contrast, Wakeman et al.[16] showed that overexpression of GDNF delivered by a viral vector into the striatum could enhance survival of human NSCs that were transplanted into the SN in the MPTP-lesioned nonhuman primate model. After 1.5 months treatment, they observed engrafted human NSC grafts distributed throughout the ipsilateral SN in parkinsonian monkeys. In addition, GDNF staining was apparent within the SN, suggesting retrograde transport of GDNF from the striatum to the SN. Madhavan et al.[17] revealed that transplantation of neural precursor cells (NPCs) elevated proliferation and neurogenesis of host NPCs in the subventricular zone, and promoted migration of newly produced neurons into the graft site in the 6-OHDA rat model. Neuroprotecton of nigrostriatal TH cells and improvement of neurobehavior had also been observed until 5 weeks after transplantation. Meanwhile, cotransplantation of more than two cell types has been actively tried to prevent immune rejection and raise regenerative effect. Berk et al.[18] demonstrated that striatal human ESC xenografts significantly survived against the host immune reaction by cotransplantation with human retinal pigment epithelial cells (hRPECs) using the 6-OHDA rat model. Until 18 days after cotransplantation, nearly 100% of human ESCs survived among the animals that received human ESC plus hRPEC cotransplants, whereas only 19.8% graft survival was observed in the animal group receiving only human ESC xenografts.

Alternative treatment options beyond cell-based therapy, ranging from neurotrophic factor gene therapy through to new drugs and deep brain stimulation, were also actively investigated for PD. Gombash et al.[19] focused on pleiotrophin (PTN) for gene therapy. They determined the size of the peak striatal PTN expression observed during rat development in order to provide a reference point for future PTN gene transfer studies. Peak PTN expression occurred 1–2 days postnatally with a mean value of about 1171 ± 312 ng/mg. Manfredsson et al.[20] demonstrated that doxycycline-mediated GDNF regulation from a single recombinant adeno-associated virus (rAAV) vector that was injected into the SN blocked weight loss, a common problem resulting from rAAV-mediated overexpression of GDNF in the nigrostriatal tract. By contrast, Marupudi et al.[21] examined the neuroprotective effect of pigment epithelium–derived growth factor (PEDF). They demonstrated that intrastriatal injection of biodegradable poly(lactic-co-glycolic acid) nanospheres containing the whole PEDF peptide protected dopaminergic neurons in the nigrostriatal tract from 6-OHDA lesioning and improved behavioral dysfunction compared to the group treated with empty nanoparticles.

Meanwhile, a number of study groups focused on a nondopaminergic medicine administered either locally or systemically as well as dietary supplementation for PD. Ma et al.[22] demonstrated that systemic administration of granulocyte-colony stimulating factor (G-CSF) and stem cell factor (SCF) prior to lesioning enhanced mobilization of endogenous NSCs in the subventricular zone to 6-OHDA–lesioned striatum, and ectopic expression of the transcription factors Nurr1 (nuclear receptor–related protein-1) and Mash1 (Mammalian achate schute Homolog 1) using viral vectors induced mobilized endogenous NSCs from the subventricular zone to differentiate into dopamine neurons in a mouse model of PD. In addition, this treatment significantly improved neurological dysfunction from 6-OHDA lesioning. Harvey et al.[23] reported that diadenosine tetraphosphate ($AP_4A$) has neuroprotective effects against methamphetamine-mediated toxicity both *in vitro* and *in vivo*. Prelesioning intracerebroventricular injection of $AP_4A$ improved locomotor activity and protected dopaminergic neurons in SN compared to the vehicle-treated group in a methamphetamine-mediated toxicity rat model. Thompson et al.[24] showed that 3,4-methylenedioxymethamphetamine (MDMA) induced dopamine neurite outgrowth, and transgelin-3 and its gene expression was reduced immediately after the cessation of MDMA treatment in a fetal rat model. These findings suggested that MDMA-induced dopamine neurite outgrowth might be mediated by transgelin-3, a protein implicated in neuronal cytoskeletal dynamics.

By contrast, dietary supplementation has been noted as an alternative therapy for PD. Pabón et al.[25] demonstrated that application of an AAV vector encoding human α-synuclein into the rats' SN induced loss of dopamine neurons in the SN, mimicking the other PD animal models. Additionally, they revealed that treatment with a spirulina diet attenuated the loss of dopaminergic neurons and prevented microglia activation compared to the control group, suggesting spirulina's anti-inflammatory action. Rehnmark et al.[26] focused on the potential benefit of a blueberry-enriched diet for the treatment of PD. They showed that tenascin-C immunoreactivity was more intense and inflow of phagocytotic microglia was faster in the blueberry-treated group than in the control group using a 6-OHDA rat model. This study indicated that tenascin level or ED-1 (CD-68)–positive microglia may be markers for recovery of dopaminergic neurons.

Deep brain stimulation (DBS) of the subthalamic nucleus (STN) has become a popular neurosurgical treatment modality for PD. Spieles-Engemann's group demonstrated that DBS in the STN of unlesioned rats increased brain-derived neurotrophic factor (BDNF) in the striatum and globus pallidus, STN's target structures, rather than GDNF[27]. Analysis of BDNF protein levels in the 6-OHDA–lesioned rats is ongoing. They also investigated the effect of STN DBS on striatal dopaminergic neurons in a 6-OHDA rat model[28]. In this study, they revealed that STN DBS halted ongoing SN dopamine neuron cell loss in 6-OHDA–lesioned rats compared to the control group. Despite this protection observed at the level of the cell body, stereological analysis of striatal TH immunoreactive neurite density and striatal dopamine levels did not show a significant difference between STN DBS and control groups. These findings suggested that STN DBS halts ongoing degeneration of SN dopamine neurons, but does not restore previously depleted dopaminergic terminals in the striatum.

The reports mentioned above relating to PD demonstrate the potential importance of growth factors such as GDNF in treating the disease, with studies showing an altered dopamine response in knock-out mice as well as the ability of GDNF to promote graft survival *in vivo*. Stem cell transplants, ranging from hUCB cells to NPCs, were shown to have some functional efficacy in animal models and the benefits of cotransplants were highlighted. The ability of certain dietary supplements to aid dopamine neuronal recovery also suggested another potential avenue for the treatment of PD. Interestingly, some clues as to why DBS is partly effective were revealed, including up-regulation of BDNF and the arresting of nigral cell death.

## ALZHEIMER'S DISEASE AND AGING

Along with PD, Alzheimer's disease (AD) is a major neurodegenerative disease with neurobehavioral and cognitive dysfunction. As with PD, aging seems to play a major role in the development of AD. A few reports explored neural pathogenesis related to aging. Bachstetter et al.[29] investigated the role of the fractalkine-CX3CR1 axis in the age-related decline in neurogenesis. CX3CR1, a chemokine receptor for the neuronally expressed ligand fractalkine, is critical for regulating microglia and blocking excessive microglia activation. They demonstrated that the loss of function of CX3CR1 in young adult rodents resulted in a significant decrease in hippocampal neurogenesis and administration of exogenous fractalkine reversed the age-related decrease in hippocampal neurogenesis. Furthermore, interleukin (IL)-1β receptor antagonist protected against the decrease in hippocampal neurogenesis induced by blocking CX3CR1 function. Further exploration of the role of microglia with respect to aging was provided by an article published in the *TheScientificWorldJOURNAL*[30]. Also, Schmitz and Chew[31] reviewed how cytokines released by microglia and oligodendrocytes could affect myelination in the CNS over time.

There were several studies that looked at microglia and the effects of growth factors that can be secreted by microglia on aging. For instance, Lee et al.[32] studied the influence of aging on the polarization of microglia. They performed intraparenchymal injections of an M1 (classical)-activating or M2 (alternative)-activating cytokine cocktail designed to elicit either a biased M1 or an M2 microglial response into the hippocampus of young and old animals. In this study, aging reduced the effectiveness of the M2 cocktail injections, but the effectiveness of the M1 cocktail inductions was increased in line with aging. Granholm et al.[33] demonstrated that nerve growth factor (NGF) levels were elevated in the hippocampus of aged rats, but reduced in the basal forebrain where cholinergic neurons degenerated similar to AD patients. They determined that this was due to the aging process blocking the response of high-affinity NGF TrkA receptors to the released NGF, rather than a reduced release of NGF itself.

The importance of mitochondrial integrity to aging was highlighted in one study by Ross et al.[34]. They showed that the lactate level in the brain and plasma, and mitochondrial dysfunction, was significantly increased in mice prematurely aged by gene mutation to a level comparable with that seen in wild-type aged controls. In this study, they used the mitochondrial DNA mutator mouse, which exhibits a three- to fourfold increase in the levels of mitochondrial DNA point mutations as well as increased levels of mitochondrial DNA deletions. This mouse has a reduced life span and fertility, weight loss, anemia, cardiac hypertrophy, and osteoporosis. The relationship between aging and mitochondrial dynamics was further explored in two manuscripts published by *TheScientificWorldJOURNAL*[35,36].

The pathogenesis of AD is still unknown and therefore is still being actively investigated. Mervis et al.[37] revealed a significant progressive loss of branching following dendritic analysis of cortical neurons that paralleled increasing cognitive deficits in the parietal cortex. The frontal cortex of patients with mild cognitive impairment possessed pyramidal neurons that manifested increased dendritic branching and spine number compared to patients with no cognitive impairment, whereas AD patients showed a significant reduction of these parameters.

Tau is a microtubule-associated protein that abnormally accumulates in a group of neurodegenerative diseases, collectively termed tauopathies, which includes AD. Heat shock protein inhibitors were demonstrated to decrease both tau and phospho-tau levels dramatically by approximately 90%, while activators led to their accumulation up to 300%[38]. Soo et al.[39] reviewed the effects of heat shock proteins with respect to neurodegenerative disorders, including AD, whereas Guglielmotto et al.[40] explored the role of oxidative stress and hypoxia to AD pathogenesis in *TheScientificWorldJOURNAL*.

A transgenic mouse model (Tg4510 mouse) was introduced at the meeting that mimicked the tauopathy observed in frontotemporal dementia with parkinsonism[41]. The impact of amyloid beta (Aβ) 1-42–derived oligomers on tau hyperphosphorylation was investigated by utilizing these models. Nguyen et al.[42] monitored the aggregation pathways of Aβ peptide by means of atomic force microscopy, hence imaging their topological feature changes (monomeric, oligomeric, and fibrillar species) over time.

Lanari and Parnetti[43] published an article in *TheScientificWorldJOURNAL* in which they tracked cerebrospinal fluid biomarkers in patients with mild cognitive impairment over 4 years and observed increased Aβ42, T-tau, and P-tau in those patients who went on to develop AD. Thal[44] discussed how impairment of the blood brain barrier could result in Aβ accumulation. Impairment of the blood brain barrier with respect to amyotrophic lateral sclerosis was a topic at the previous year's ASNTR meeting[45].

With respect to supportive therapy for the aging process, Acosta et al.[46] reported on the beneficial role of dietary supplementation (NT020; Neutrastem; Naturatherapeutics, FL) against age-related deficits in learning and memory. NT020 is a combination of nutrients that were found in natural compounds such as blueberry, green tea, and carnosine. Oral administration of NT020 ameliorated cognitive deficits found in aged rats in a water maze behavioral test. Asha Devi[47] looked at dietary means and exercise to modify the effects of aging, whereas Kulkarni et al.[48] looked at the benefits of the natural compound curcumin as an antidepressant in reviews published in *TheScientificWorldJOURNAL*.

By contrast, Park et al.[49] investigated endogenous neuronal proliferation induced by transplanted human NSCs. They demonstrated that transplantation of fetal NSCs (ReN001; ReNeuron, U.K.) into the lateral ventricle promoted proliferation of endogenous neurogenitors stained with doublecortin and bromodeoxyuridine in the dentate gyrus of aged rats.

A number of study groups investigated new treatment strategies for AD. Li et al.[50] developed a new bacteriophage-based Aβ peptide vaccine. They immunized amyloid precursor protein (APP) transgenic mice at the age of 9 months with either N-terminal Aβ or C-terminal Aβ peptide fragments conjugated to the QB bacteriophage without using an adjuvant. They found that both vaccines induced a sufficiently robust antibody response to reduce Aβ deposits in the brain of APP transgenic mice with neither microglia activation that would lead to an inflammatory response nor microhemorrhage. Alternative means to reduce Aβ deposition were shown by a number of groups. Sanchez-Ramos et al.[51] presented data revealing that administration of G-CSF to AD transgenic mice reversed cognitive performance and significantly decreased Aβ deposition in the hippocampus and entorhinal cortex. G-CSF–treated transgenic mice were also found to exhibit a significant increase in total microgliosis and increased synaptophysin immunostaining in hippocampal CA1 and CA3 regions. Smith et al.[52] showed that feeding of a standardized turmeric extract, HSS-888, to AD mice significantly reduced brain levels of soluble and insoluble Aβ, as well as phosphorylated tau protein. In addition, primary cultures of splenocytes from these mice exhibited enhanced cellular immunity (increased IL-4 to IL-2 ratio). Zhu et al.[53] presented a remarkable reduction of Aβ levels/Aβ plaques and associated inflammation in AD transgenic mice following strain-matched wild-type bone marrow infusion, whereas in *TheScientificWorldJOURNAL*, Solomon[54] reviewed other potential immunotherapies for the treatment of AD. Bergamaschini et al.[55] reviewed whether the immunomodulatory effects of heparin could influence Aβ levels.

The above reports again highlight the potential benefits of dietary supplementation for the treatment of AD and modification of the effects of aging. In addition, the potential roles of growth factors, microglia, and inflammation are being intensely studied, providing clues to understanding the processes involved in aging and AD. Therapies that reduce Aβ accumulation are also becoming a major focus of research.

## STROKE

The only FDA-approved treatment for stroke is the use of the thrombolytic agent, tissue-plasminogen activator. This therapy is very limited due to a narrow therapeutic window and the risk of hemorrhagic complications. These characteristics of stroke should be taken under consideration when developing an alternative treatment strategy such as cell-based therapy. A number of studies have investigated cell-based therapies and other related factors for stroke.

By contrast to PD studies, investigations focusing on transplantation with various types of stem cells to treat stroke were prevalent. Daadi et al.[56] showed the potential of human ESC-derived NSCs in a neonatal brain hypoxia animal model. They demonstrated that transplantation of these NSCs into the forebrain of rats with hypoxia-ischemia significantly improved neurological deficits and promoted axonal sprouting in the stroke-damaged brain compared to the vehicle group. Furthermore, grafted animals showed a significant decrease in the $CD3^+$, $CD4^+$ T cells, suggesting an anti-inflammatory effect. Gorenkova et al.[57] demonstrated the potential of NSC-endothelial cell incorporation in a cell-based therapy for stroke. They injected polyethylene glycol (PEG)–based hydrogel bead–encapsulated NSCs and endothelial progenitors into the stroke cavity. After 7 days, they found transplanted endothelial and neural stem cells migrating from the hydrogel beads within the lesion cavity. Some integration with host tissue and primitive organization of the endothelial cells into tubular structures was also observed.

hUCB stem cells are being heavily researched with an eye towards their potential capability as a cell-based therapy for stroke. Shahaduzzaman et al.[58] demonstrated that there was a significant difference in gene expression from neuronal cells induced by coculture with hUCB cells under hypoxic conditions compared with normoxic conditions. Furthermore, a cell viability analysis revealed an increase in the viability of neurons cocultured with hUCB compared to monocultured neurons under exposure to oxygen-glucose-deprived environment. Womble et al.[59] showed that transplantation of hUCB cells from which the monocyte/macrophage subpopulation ($CD14^+$) had been depleted failed to improve the neurological outcome to the same extent as transplantation of the other fractions of UCB cells (T-cell depleted, B-cell depleted, $CD133^+$-depleted, and whole mononuclear fraction) in the middle cerebral artery occlusion (MCAo) rat model of stroke. Furthermore, depletion of $CD133^+$ and $CD14^+$ cells resulted in infarct volumes and motor function comparable to that of the MCAo only group.

Commercial stem cells for stroke therapy were also reported at this year's conference. Hess et al.[60] reported the potential of MultiStem® (Athersys, OH), a proprietary adult adherent stem cell clinical product in multiple preclinical animal disease models, including stroke. They also introduced the proposed design of their phase I trial for this preparation against clinical stroke. By contrast, Sinden[61] presented a newly developed clonal stem cell line, ReN001 (also see Eve et al.[62] and Park et al.[49]), which is derived from human fetal cortex and has shown potential as a stroke treatment, leading to approval in the U.K. for use in a clinical trial against stroke. Musso et al.[63] reported on the long-term effects on cerebral blood flow, as measured by laser Doppler, in MCAo rats treated with the ReN001 cell line. They found that cerebral blood flow on the lesioned side of the cell-treated group improved compared to the untreated rats and demonstrated a similar oscillatory nature as that observed in sham-lesioned animals.

A number of medicines to protect endangered neural tissues or prevent apoptosis of neural progenitors following stroke were also investigated. Luo et al.[64] demonstrated the ability of pifithrin-α, a p53 inhibitor, to enhance the survival of endogenous neural progenitor cells originating from the subventricular zone. They found that pifithrin-α administration to rats 6–9 days after MCAo enhanced functional recovery, and improved survival, proliferation, and migration of subventricular zone progenitors, suggesting increased neurogenesis. Neurotrophic factor also has been applied with gene-transfected viral vectors for stroke. Mesencephalic astrocyte-derived neurotrophic factor (MANF) is a secreted protein that reduces endoplasmic reticulum stress. Airavaara et al.[65] demonstrated the putative role of MANF against cerebral ischemia. They found that AAV-MANF pretreatment in the cortex significantly decreased the infarction volume and improved the neurological score in the MCAo rodent model.

Bacigaluppi and Hermann[66] reviewed potential neuroprotective targets for the treatment of stroke in *TheScientificWorldJOURNAL*, whereas three studies[67,68,69] investigated the neuroprotective effects of erythropoietin as a clinical means to treat stroke or elucidation of its potential mechanisms. This was a topic of interest reported at the previous ASNTR meeting[45].

Reports at the ASNTR meeting provide hope for the eventual successful treatment of stroke with the announcement of two potential commercial stem cell products (Multistem and ReN001) and we await the results of clinical trials using these products with anticipation. Additional studies demonstrating some degree of benefit with hUCB and NSC transplants, relating to alterations in the immune response, growth factors, and endogenous proliferation, provide us with hope that the field is working towards the successful treatment of strokes.

## SPINAL CORD INJURY, AMYOTROPHIC LATERAL SCLEROSIS, AND PAIN

Spinal cord injury (SCI) is not a common disease, but it can affect relatively young people who often become a huge burden to themselves and society due to high medical costs throughout their lives. Currently, there is no effective treatment for SCI except high-dose methylprednisolone, which has many critical side effects and, consequently, irreversible disability of motor and sensory activity may remain. Therefore, most studies have focused on the development of new treatment strategies. Steuer and Guertin[70] reviewed the developments made in SCI research using mice in 2008 in *TheScientificWorldJOURNAL*, whereas more recent studies are highlighted at the ASNTR meeting as outlined below.

Bobek et al.[71] demonstrated the role of vascular endothelial growth factor (VEGF) and NGF in the treatment of SCI. They administered cDNA plasmids of VEGF and NGF into an experimental mouse model of SCI. They found that there were neither behavioral differences between tested animals following VEGF and NGF plasmid administration nor changes in their overall gene expression or immunohistochemical staining. Khaing et al.[72] investigated the role of high-molecular-weight hyaluronic acid (HA), a major component of the native extracellular matrix of the CNS in glial scar formation after SCI. They demonstrated that HA implantation into an injured spinal cord decreased astrogliosis and the astroglial scar component in and around the injury site.

In contrast, a number of groups applied stem cell–based therapy for the treatment of SCI. Ghosh et al.[73] investigated polysialyl transferase expression of Schwann cells (PST-SC). This produces cell surface polysialic acid (PSA) residues on the neural cell adhesion molecule that reduces cell-cell interactions. They found significant rostrocaudal migration of the transplanted PST-SCs into the SCI epicenter between 2 and 5 mm from the implantation/lesion site compared to the control Schwann cells, which remained confined to the injury site. Furthermore, there was extensive corticospinal axon growth within and surrounding the implantation site only with PSA-modified Schwann cells and significant improvement of locomotor performance in PST-SC–implanted animals. In contrast, Jain et al.[74] investigated the effect of Schwann cell transplantation on neuropathic pain in SCI. There has been a raised concern that transplantation of Schwann cells may enhance sensory plasticity and thus contribute to and enhance neuropathic pain. They found that there were no significant changes in the thermal hyperalgesia and cutaneous allodynia responses between injury only and injury with Schwann cell transplant groups in chronically injured spinal cords. Golshani et al.[75] studied transplantation with human fetal spinal cord–derived NPCs for SCI. In this study, they demonstrated that implantation of NPCs either into or adjacent to the injury site led to good survival, proliferation, and rostrocaudal migration. Moreover, the concurrent administration of FK506, an immunosuppressant, with NPC implants provided a significant, approximately twofold, enhancement in NPC survival and proliferation, as well as extending their migratory distance. Priest et al.[76] introduced another commercialized stem cell line, GRNOPC1 (Geron, CA), that contains human ESC-derived oligodendrocyte progenitors. They showed that administration of GRNOPC1 into the contusion site 7 days after a thoracic SCI in rats improved hindlimb locomotor activity and reduced parenchymal cavitation at the injury site 9 months post-transplantation. This cell line has been approved by the FDA for phase I clinical trials in the treatment of SCI. Teng et al.[77] investigated the effect of SCI-induced astrogliosis on NSC engraftment using glial fibrillary acidic protein (GFAP) and vimentin knock-out mice. This transgenic mouse model limited the degree of post-SCI reactive astrogliosis. They found that neurobehavioral recovery was significantly higher in the human NSC-transplanted knock-out mice compared with those of the wild-type mice that underwent the same quality of human NSC-polymer placement.

A new means of measuring locomotor recovery, the CatWalk™ device (Noldus Inc, Netherlands), was reviewed at the ASNTR meeting[78]. This device works by recording the time and location of an animal’s footprints as it transverses an illuminated glass walkway. They analyzed cervical cord–injured rodents’ locomotion using this equipment and discussed some of the pitfalls of the device.

Amyotrophic lateral sclerosis (ALS), a fatal progressive neurodegenerative disease caused by the degeneration of motor neurons, has neither an effective treatment nor a complete understanding of its cause. Stoner et al.[79] reported on a significant decrease of microvascular density in the gray matter of the spinal cord of ALS mice at both early and late stages, compared to the control group. These findings suggested that ALS motor neuron degeneration may be due to the accumulation of metabolic waste and by-products, leading to an increasingly excitotoxic microenvironment for motor neurons. Woods et al.[80] found that circulating endothelial cells significantly decreased in the peripheral blood of ALS patients with moderate or severe disease compared to controls. The reduction of endothelial cells in the peripheral blood of ALS patients suggested impairment of endothelial cell replacement during the course of disease, possibly leading to blood/spinal cord barrier dysfunction in ALS.

A few investigations focusing on stem cell–based therapies were reported at the 2009 ASNTR meeting. Federici et al.[81] introduced another commercialized stem cell line, NSI-566RSC (NeuralStem, Inc., MD), derived from human fetal spinal cord for spinal cord transplant therapy. This cell line has been shown to prolong survival in rodent ALS models. In this study, they showed that multiple unilateral injections of NSI-566RSC into the spinal cord of minipigs were safe and the grafts survived for 4 weeks after transplantation. Miller et al.[82] showed that $25 \times 10^6$ mononuclear hUCB cells was the most effective dose for anti-inflammatory action in line with previous studies that showed that this dose was the most effective in increasing mouse life span and delaying disease progression in an ALS mouse model. Microglia density in the gray matter of the ventral horn region of the spinal cord in ALS mice decreased significantly after intravenous injection of mononuclear hUCB cells with $25 \times 10^6$ dose, compared to 10 or $50 \times 10^6$.

A few papers focused on neuropathic pain. Collado et al.[83] studied intrathecal injection of DNA-encoding ser-histogranin, a synthetic equivalent of the naturally occurring peptide N-methyl-D-aspartic acid receptor antagonist, found in the adrenal chromaffin cells, in a rat pain model. They found that ser-histogranin attenuated the formalin-evoked pain-related flinching behavior. Another study showed that transplantation of predifferentiated GABA (gamma aminobutyric acid) -ergic NPCs or MASH1-transduced fibroblast growth factor (FGF)-2–deprived neurospheres attenuated neuropathic pain in a rat neuropathic pain model[84]. This finding suggested that intraparenchymal grafts of GABAergic NPCs may have a potential to integrate and restore spinal inhibitory neural circuitry for long-term pain alleviation.

The reporting of commercial stem cell preparations for the treatment of SCI (GRNOPC1) and ALS (or also SCI; NSI-566RSC) provides hope for finding treatments for these disorders. The results of clinical trials with these compounds are awaited with great interest. The use of a number of other stem cell preparations provides hope for alternative preparations in the future if the two commercial products featured do not prove successful. The potential treatment of long-term pain by stem cell transplants was also featured. However, the lack of any behavioral benefit following VEGF or NGF plasmid insertion for the treatment of SCI is rather disappointing and the reasons for this lack of any functional effect requires further clarification.

## HUNTINGTON'S DISEASE

Huntington's disease (HD) is a slowly progressive and autosomal-dominant inherited neurodegenerative disorder. Comparison of the concentrations of 17 striatal metabolites between normal mice and the R6/2 mice (HD transgenic mice) by *in vivo* $^1$H proton nuclear magnetic resonance spectroscopy performed at 8 and 12 weeks of age revealed that the creatine (Cr) + phosphocreatine variable by itself was by far the most significant metabolite[85], highlighting the potential importance of mitochondrial dysfunction in this disorder.

A few researchers investigated cell-based treatment modalities for HD. Rossignol et al.[86] investigated the potential treatment of bone marrow mesenchymal stem cells (BMSC) in the 3-nitropropionic acid rat model of HD. They demonstrated that intrastriatal injection of BMSCs improved motor dysfunction modestly, and those transplanted cells were still viable and metabolically active 71 days post-transplantation. They suggested that the observed recovery of function was attributed to the release of trophic factors from the MSCs because very few MSCs showed evidence of transdifferentiation. Johansson et al.[87] demonstrated that differential graft survival of two human NSC lines in a rat model of HD was not due to a difference in major histocompatibility complex (MHC) expression, but mainly due to a difference in their propensity to induce a lymphocyte proliferation through the Fas system. The shortest surviving cell line elicited strong infiltration of lymphocytes resulting from a fourfold higher expression of Fas ligand (FasL), even though neither cell line expressed MHC antigens or complement *in vivo*.

The fate of striatal grafts in patients with HD was explored by Cicchetti et al.[88]. Embryonic striatal grafts without genetic HD markers were reported to experience robust pathological changes that were the same as those observed in HD. In particular, medium spiny neurons were observed within the genetically unrelated grafts that exhibited pronounced morphological changes in comparison with interneurons. This is a similar report to those presented at the previous year's meeting related to PD and striatal grafts[45]. In contrast to the studies on ways to treat HD presented at the meeting, *TheScientificWorldJOURNAL* published a review of the involvement of the ubiquitin-proteasome pathway in the disease[89].

Evidence of mitochondrial dysfunction in HD, as exemplified by the increased presence of creatine and phosphocreatine in animal models of the disease, helps to provide further clues as to how the presence of too many trinucleotide repeats in the huntingtin protein may cause the disease. The observation of disease pathology in grafted tissue in HD, similar to a finding reported last year for PD provides an important insight into the disease itself. The relatively modest beneficial effect and survival of stem cell transplants in HD animal models provides some hope for a future cure.

## TRAUMATIC BRAIN INJURY AND OTHER NEURODEGENERATIVE DISORDERS

Moradian et al.[90] evaluated dendritic parameters using a closed-head model of minimal traumatic brain injury (mTBI). They found that two mTBI episodes resulted in a reduced spine density in both apical and basilar trees, and three mTBI episodes or a single TBI episode equivalent to the sum of three mTBI episodes was not associated with spine loss, but by an increase in spines. Yu et al.[91] developed TBI animal models encompassing three levels of disease severity (mild, moderate, and severe) using the controlled cortical impact injury technique. They demonstrated that abnormalites in locomotor and cognitive behaviors in TBI-exposed rodents were dependent on TBI severity.

Garbuzova-Davis et al.[92] reported on their investigation into hUCB treatment for Sanfilippo syndrome type B (mucopolysaccharidosis [MPS] III B). They demonstrated that intravenous or intraventricular injection of mononuclear hUCB cells improved behavioral dysfunction in a MPS III B mouse model. Monocyte/macrophage cells from hUCB were found to play a major role in these benefits.

The effects of grafting hippocampal stem/progenitor cells into the hippocampi of rats shortly after status epilepticus for restraining chronic epilepsy development was reported by Kuruba et al.[93]. Overall, status epilepticus rats with grafts exhibited 50–62% reductions in seizure frequency and intensity, compared to those with sham surgery. Rachubinski et al.[94] investigated the role of NPC transplantation at the neonatal stage before postnatal neurodegenerative changes for Down syndrome. They transplanted NPCs in the hippocampus of transgenic Down syndrome mice on postnatal day 2. Transplantation of NPCs improved a righting response and moderately shortened the time to eye opening compared to the vehicle group. Vorobyeva et al.[95] investigated the effects of transplanting human BMSCs or rat embryonic neuronal tissues into a rat model of multiple sclerosis. They found that BMSCs without retinoic treatment stimulated movement activity in the short term, failing to re-establish muscle tone, whereas retinoic-treated BMSCs restored muscle function in accordance with the absence of proliferative processes in immunocompetent organs. Furthermore, embryonic neuronal tissue grafts in the sensorimotor cortex and subcutaneous area also restored movement activity. Most treatment modalities brought normalized phospholipid levels correlating with high levels of myelin in blood plasma. Walczak et al.[96] introduced high-resolution magnetic resonance cellular imaging to monitor the delivery and acquire information about the position of grafted cells. They used this method for the *in vivo* imaging of grafted glial-restricted precursor cells into rodent brains for the treatment of myelin disease.

The neurodegenerative effects of drugs of abuse were also studied. As previously mentioned, the ability of Ecstasy (MDMA) to affect dopamine neuron growth was reported by Thompson et al.[24]. Lee et al.[97] determined that cocaine caused NPCs to accumulate reactive oxygen species due to N-oxidation of cocaine via cytochrome P450, and subsequently inhibited proliferation of NPCs by down-regulation of cyclin A2 in developing fetal rat brains.

Of interest, Manfredsson et al.[98] compared the weight loss effect of GDNF injection into the hypothalamus and SN using GDNF gene-transfected viral vectors. They found that the extent of weight loss was much greater in the SN-GDNF animals compared to the hypothalamus-GDNF animals. Moreover, they found a known downstream protein of GDNF signaling, phosphorylated extracellular signal-regulated kinase, to be expressed in the paraventricular hypothalamic area. Parihar et al.[99] investigated the role of chronic mild stress on endogenous neurogenesis. They found a 51% increase in the production of new cells/day (bromodeoxyuridine positive) and a 53% increase in the numbers of newly born neurons (doublecortin positive) in the subgranular zone-granular cell layer of rats exposed to predictable chronic mild stress (daily 5 min of restraint stress), in comparison to age-matched naïve rats.

Stem cell transplantation may have some beneficial effects against a number of different neurodegenerative disorders, including Sanfilippo syndrome, multiple sclerosis, and epilepsy, as well as the disorders featured in earlier sections. However, this form of treatment is still in its infancy and will require further study before its full benefits can be determined. The development of an animal model for different degrees of TBI provides hope for the elucidation of future treatments. The ability of routine chronic stress to boost endogenous stem cell production raises interesting questions about the role of stress, and the development and potential treatments for disorders that would respond well to boosting endogenous stem cell activity.

## CELL CHARACTERIZATION AND NORMAL BIOLOGICAL MECHANISM

Many investigators have studied the characterization of cells and tissues for regenerative medicine. Baek et al.[100] reported that hippocampal NSCs derived from the adult rat brain are insulin dependent for proliferation and undergo a nonapoptotic, autophagic cell death following insulin withdrawal. Insulin-deprived hippocampal NSCs exhibited morphological and biochemical markers of autophagy, including massive formation of autophagic vacuoles, accumulation of Beclin 1, and the type II form of microtubule-associated protein 1 light chain 3 without evidence of apoptosis. Bernal and Peterson[101] studied the colocalization of GFAP and the VEGF receptor Flk1 on NSCs. They demonstrated that Flk1 was expressed in a small population of GFAP-positive cells that also are known to express VEGF. This population of GFAP-positive cells was also Sox2 positive, indicating by this combination of expression that these cells are type 1 NSCs. Eve et al.[62] investigated the ability of CTX0E03 cells (the ReN001 cell line) to promote angiogenesis. They found that CTX0E03 secreted VEGF, endostatin, and tissue inhibitor of metalloproteinases using enzyme-linked immunosorbent assay analysis. They also demonstrated that there was an optimal concentration of VEGF with respect to the average distance of vascular-like projections from an aortic ring in an *in vitro* rat aorta angiogenesis assay. Wang et al.[102] investigated the mechanisms of NSC-mediated T-cell suppression. They found that high levels of nitric oxide (NO) and prostaglandin $E_2$ ($PGE_2$) were induced in the T cells when cocultured with NSCs. T-cell proliferation inhibited by coculture with the NSCs was significantly restored by inhibitors of NO and $PGE_2$. This suggested that NSCs appear to suppress T cells, at least in part, by the induction of NO and $PGE_2$. Of interest, Hayashi et al.[103] studied the expression of two discrete types of melatonin receptors and their role in proliferation and differentiation of cultured human amniotic epithelial cells. Melatonin dose dependently suppressed proliferation, but enhanced neural differentiation of melatonin receptor 1–expressing amniotic epithelial cells. Dentelli and Brizzi[104] expanded on the ability of the microenvironment to influence stem cell proliferation in an article in the *TheScientificWorldJOURNAL*.

A number of studies focused on the identification of biological properties of biomaterials such as viral vectors and biochemicals. Dodiya et al.[105] demonstrated that AAV serotype 1 and 5 were superior to type 8 for gene delivery to the nonhuman primate striatum. Emborg et al.[106] tested the ability of pioglitazone to penetrate cerebrospinal fluid. Pioglitazone, a thiazoledionedione currently FDA approved as an antidiabetic treatment, has been proposed as a neuroprotective therapy. They demonstrated that oral administration of 5 mg/kg in rhesus monkeys was an effective dose to induce detectable higher levels of pioglitazone in plasma and cerebrospinal fluid consistently. Fortress et al.[107] demonstrated that pro-NGF induced up-regulation of p75, the low-affinity NGF receptor, and sortilin in the basal forebrain of aged rats, whereas systemic administration of NGF increased the expression of the high-affinity NGF receptor, trkA, in the basal forebrain and enhanced memory performance. These findings suggested that an alternative receptor activation by pro-NGF may contribute to the basal forebrain cholinergic neuron degeneration that has been demonstrated in the aged rodent brain.

A few groups investigated the molecular biology for neuroregenerative phenomenon. Macklis[108] reviewed molecular controls over differentiation, connectivity, and survival of specific neuronal lineages. They demonstrated that new neurons could be added to adult neocortical circuitry via manipulation of transplanted or endogenous precursors *in situ* (e.g., corticospinal motor neurons), indicating that cellular repair of cortical and cortical output circuitry is possible, if controls over specific lineage differentiation are understood. By contrast, Maksoud[109] reviewed strategies to enhance the regenerative capacity of the CNS. They included molecular, cellular, and axonal guidance approaches that enhance one or more of the following cellular and histological processes: neurogenesis, synaptogenesis, angiogenesis, and axonal and dendritic outgrowth.

The characterization of stem cells, with respect to the presence of receptors and their normal proliferative function, is of considerable importance. The presence of receptors for growth factors such as GFAP and VEGF reveals that the cells can respond to these factors themselves as well as being able to secrete them. The ability of NSCs to inhibit T cells via NO and $PGE_2$ provides a possible mechanism by which stem cells can modulate the immune response. The dependence of hippocampal stem cell proliferation and survival on insulin raises important questions about the optimal conditions for cell growth. In line with previous meetings, the use of viral vectors and small molecules to induce specific characteristics in stem cells is of considerable importance.

## CELL PREPARATION

A number of reports focused on cell preparations, including their isolation, differentiation, and proliferation. In the *TheScientificWorldJOURNAL,* Petrovic and Stefanovic[110] reviewed the potential for obtaining stem cells from dental tissue, whereas Deb[111] explored ways to store embryonic stem cells. At the meeting, one group studied the effects of Cr on survival, migration, and differentiation of mouse and human NSCs[112]. They found that chronic Cr exposure to both mouse and human NSCs resulted in a dose-dependent increase in neurosphere size and total cell numbers, and an improved migratory potential of NSCs. Furthermore, Cr exposure also promoted the differentiation of NSCs towards the neuronal lineage, particularly supporting the GABAergic phenotype. The effects of high (20%) and low (3%) oxygen-tension culturing in conjunction with FGF-2 and FGF-8 on the expansion of precursor cells was reported by Jensen et al.[113]. They found that the combination of FGF-2 treatment at low oxygen and exposure to high oxygen tension during differentiation provided the most effective numerical expansion of fetal mesencephalic precursor cells. Another group optimized the properties of photocross-linkable, HA-based hydrogels to encourage differentiation of ventral midbrain progenitors into dopamine neurons[114]. They used high-molecular-weight HA and modified it with methacrylic anhydride for hydrogels. A higher degree of methacrylation resulted in decreased swelling ratios and degradation rates, indicating more tightly cross-linked hydrogel networks. Therefore, they could control the mechanical properties of a 3D culture environment within HA-based hydrogels (softer vs. harder). Marschinke et al.[115] investigated dopamine nerve fiber growth in fetal VM culture. They first observed a long-distance outgrowth in the absence of glial cells that disappeared over longer time points. Second, long-term dopamine nerve fiber outgrowth was observed from 5 to 7 days *in vitro* under the guidance of glial cells. The integrin-associated protein CD47 is expressed in all tissue and serves as a ligand for the signal regulatory protein Sirpα, which is a promoter for migration and synaptogenesis. They found that nonglial-guided dopamine nerve fiber outgrowth in CD47 knock-out mice reached significantly longer distances from the tissue slices compared to CD47-positive mice after 14 divisions.

Meanwhile, Piao and Zhao[116] demonstrated that SCF and G-CSF promoted neuronal fate determination and differentiation of NSCs. When SCF and G-CSF were added to NSCs during NSC differentiation, they found that the number of TuJ1-positive neurons was increased, and the number of GFAP-positive astrocytes was decreased by SCF and G-CSF.

The rapidly progressing field of induced pluripotent stem cells (iPS cells) was covered by Singec and Snyder[117], who reported their experience with the generation and differentiation of iPS cells into specific neural lineages, such as dopamine neurons and motor neurons. They successfully generated iPS cells from human fibroblasts derived from normal individuals and from various patients with genetic disorders by retroviral delivery of transcription factors. Vazin et al. identified chemical factors that are responsible for stromal-derived inducing activity (SDIA) by performing gene expression profiling of PA6 cells and comparing these cells with other cell lines that lack SDIA[118]. SDIA refers to the property of (mouse) stromal cell lines, such as PA6, to induce ESCs to differentiate into dopaminergic neurons. In this study, they observed a novel combination of four factors, stromal cell–derived factor 1, PTN, insulin-like growth factor 2, and ephrin B1, which they have termed SPIE. Worden et al.[119] examined the differences between three normal human ESC lines and an abnormal line in their ability to differentiate to midbrain dopamine neurons using a chemically defined, feeder-free system. The abnormal line displayed an enhanced degree and speed of differentiation relative to all the normal lines, while the speed and degree of differentiation of the BG03 line was intermediate between the other normal cell lines and the abnormal line. These findings suggested that the ability of stem cells to differentiate is influenced by karyotypic abnormality, as well as by other genetic differences between human ESC lines.

Different ways to promote the survival and differentiation of stem cells were reported, including use of specific growth factors such as the SPIE cocktail for dopaminergic differentiation. The observation that the speed and degree of differentiation of ESCs was influenced by karyotypic abnormalities does raise interesting questions about the prolonged culture of cells, which increases the likelihood of obtaining chromosomal mutations.

## INTERESTING OTHER TOPICS

Freeman and Granholm[120] tested the detrimental effects of a high fat and high cholesterol diet. They grafted hippocampal tissue from embryonic rats to the anterior eye chamber of host animals that were fed a high fat and high cholesterol diet for 6 weeks. The high fat and cholesterol diet led to a marked reduction in hippocampal transplant growth, and this effect was blocked by IL-1 inhibitor. By comparison, reviews by Panza et al.[121] and Solfrizzi et al.[122], published in *TheScientificWorldJOURNAL*, looked at the link between dietary fatty acids and dementia, whereas Reiss and Wirkowski[123] reported on the potential benefit of lipid-lowering drugs for the treatment of neurological disorders..

Bjugstad et al.[124] assessed the biocompatibility of PEG-based hydrogels implanted into the brains of healthy rats and evaluated the release of growth factors from the hydrogels over a 2-month period. They tested PEG strands that were engineered to degrade relatively fast, slow, or not at all (nondegradable). In this study, biocompatibility of PEG-based hydrogels was partially dependent on the rate of degradation in the brain, with faster degradation rates associated with greater immunoreactivity, e.g., GDNF was released from the slow-degraded hydrogel for 2 months after implant, with increased levels of GDNF up to 50 μm from the implant site. A novel method for tracking cells after transplantation was also reported[125]. A mouse NPC line harboring a triple fusion reporter gene encoding a luciferase, a red fluorescent protein, and a thymidine kinase positron emission tomography (PET) reporter gene was created. NPCs were also transfected with super paramagnetic iron oxide labeling for magnetic resonance imaging (MRI) to create a clinically applicable system to monitor NSCs after transplantation. They obtained high spatial resolution from MRI and high sensitivity with PET for the tracking of NPCs.

As reported in other sections, diet can have important effects on cell viability, and a high fat and high cholesterol diet can disrupt the grafting of transplanted tissue. The importance of what you eat requires further elucidation with respect to potential treatments. In addition the use of hydrogels as a growth factor delivery system could be used to treat a number of different disorders. A clinically effective mechanism to track cells after transplantation is also of great importance.

In addition to the presentations, there were several discussions relating to important topics in the field, including stem cells proceeding to clinical trials without adequate large animal testing. The perils of “medical tourism” and the NIH announcement of changes to their guidelines for human stem cell research were also discussed and resulted in the issuance of a press release reporting on the ASNTR stance on these changes.

## CONCLUSIONS

Evan Snyder, ASNTR president 2008–2009, characterized the annual meeting of the ASNTR during the presidential symposium as “[the] ASNTR has always been at the forefront of neural repair, starting with its early focus on transplantation and [is] now broadening its scope to embrace all means of restoring function to the dysfunctional or maldeveloped central nervous system”[126]. The number of studies that focus on issues beyond simple transplantation have increased. Many investigators have explored more effective and safer methods for regenerative medicine, such as a combination therapy of cell transplantation and medicine. More advanced techniques and devices for research and clinical trial have been developed. In addition, new sources of stem cells have emerged, such as iPS cells. Apparently, these different research fields for neuroregenerative medicine appear to be individual and independent. However, eventually they should come together and become unified in order to be applied to real patients with intractable neurodegeneration. We believe that the ASNTR meeting and its perspective will help to unify the multiple fields into one goal, regeneration of the CNS.
